# Wnt Signalling in Acute Myeloid Leukaemia

**DOI:** 10.3390/cells8111403

**Published:** 2019-11-07

**Authors:** Alicja M. Gruszka, Debora Valli, Myriam Alcalay

**Affiliations:** 1 Department of Experimental Oncology, Istituto Europeo di Oncologia IRCCS, Via Adamello 16, 20 139 Milan, Italy; debora.valli@ieo.it (D.V.); myriam.alcalay@ieo.it (M.A.); 2 Department of Oncology and Hemato-Oncology, University of Milan, Via Festa del Perdono 7, 20 122 Milan, Italy

**Keywords:** acute myeloid leukaemia, Wnt signalling, prognosis, treatment

## Abstract

Acute myeloid leukaemia (AML) is a group of malignant diseases of the haematopoietic system. AML occurs as the result of mutations in haematopoietic stem/progenitor cells, which upregulate Wnt signalling through a variety of mechanisms. Other mechanisms of Wnt activation in AML have been described such as Wnt antagonist inactivation through promoter methylation. Wnt signalling is necessary for the maintenance of leukaemic stem cells. Several molecules involved in or modulating Wnt signalling have a prognostic value in AML. These include: β-catenin, LEF-1, phosphorylated-GSK3β, PSMD2, PPARD, XPNPEP, sFRP2, RUNX1, AXIN2, PCDH17, CXXC5, LLGL1 and PTK7. Targeting Wnt signalling for tumour eradication is an approach that is being explored in haematological and solid tumours. A number of preclinical studies confirms its feasibility, albeit, so far no reliable clinical trial data are available to prove its utility and efficacy.

## 1. Introduction

Acute myeloid leukaemia (AML) constitutes a heterogeneous group of clonal haematopoietic malignant disorders in which failure to differentiate and over-proliferation in the stem cell/progenitor compartment result in accumulation of non-functional myeloblasts known as blasts [1]. The morphological heterogeneity of AML is underlain by the diversity of genotypes encountered [2].

Non-random chromosomal abnormalities are present in approximately 52% of all adult primary AML patients and have long been considered the genetic events that cause and drive the neoplasm [1]. The four most common rearrangements in AML include: t(15;17), t(8;21), inv(16) and translocations involving the 11q23 locus and have frequencies between 3% and 10%. In AML, translocations give rise to gene fusions that encode specific oncogenic chimeric proteins, often acting as aberrant transcription factors. The translocations listed above encode promyelocytic leukaemia/retinoid receptor alpha (PML/RARα), acute myeloid leukaemia 1/eight-twenty-one (AML1/ETO), subunit B of core binding factor/myosin heavy chain 11 (CBFB/MYH11), and numerous mixed lineage leukaemia (MLL)-oncofusion proteins, respectively [1]. By contrast, about 40–50% of all AML cases presents with cytogenetically normal blast karyotype when assessed by conventional methods [3]. Although this group has an intermediate risk of relapse, a huge heterogeneity in terms of clinical outcome occurs in this population [2]. In recent years, significant advances have been made in defining genomic variations in normal karyotype AML patients, with the mutation status of nucleophosmin (*NPM1*), Fms-like tyrosine kinase 3 (*FLT3*), CCAAT/enhancer-binding protein alpha (*CEBPA*), isocitrate dehydrogenase 1/2 (*IDH1/2*), TET methylcytosine dioxygenase 2 (*TET2*) genes, etc. shown to be relevant [2,3]. Genes mutated in AML fall into several functional categories: transcription factors (e.g., oncofusions, mutated *AML1*), signalling genes (e.g., *FLT3-ITD*, mutated *c-KIT*), DNA-methylation-associated genes (e.g., DNA methyltransferase 3A gene (*DMNT3A*), *IDH1/2*, *TET2*), chromatin-modifying genes (e.g., *MLL*-oncofusions), molecular chaperones (*NPM1*), tumour suppressor genes (e.g., *TP53*), and spliceosome- and cohesion-complex genes [4]. Both, networks controlling normal haematopoiesis (RAF/MEK/ERK, JAK/STAT, PI3K/AKT/NFKB) and evolutionary conserved pathways (Notch, Wnt or Hedgehog) are deregulated or hijacked by the neoplastic clones [5].

In AML, there is a hierarchical cellular organization, somewhat analogous to normal haematopoiesis [6]. Only a minor fraction of self-renewing leukaemic stem cells (LSCs) at the apex of this hierarchy can maintain the disease long-term [6]. From the perspective of a successful treatment, the cancer stem cell model implies that in order to eradicate the disease and achieve long-term remissions, the LSC population must be eliminated [6]. Notably, Notch-, Wnt- and Hedgehog-signalling pathways, are of particular importance to the LSC biology [5].

Here we review the role of Wnt signalling in the pathogenesis of acute myeloid leukaemia (AML) and analyse its potential as a therapeutic target for the treatment of AML. Although distinct from other AML subtypes in terms of biology and therapy, for the purpose of this review t(15;17)-bearing acute promyelocytic leukaemia (APL) has not been separated from the other subgroups.

## 2. Wnt-Signalling Pathway

Wnt signalling constitutes a group of signal transduction pathways involved in development. In particular, Wnt signalling is important for the morphogenesis and cellular movements at the basis of organ formation. The name Wnt is a portmanteau made up of the words: Wingless and Int-1, denoting the phenotype identified in the fruit fly with Wnt1 mutations and the site of viral integration linked to carcinogenesis, respectively [7]. Signalling by the Wnt family of secreted glycolipoproteins controls embryogenesis and organogenesis, but is inactive in most adult tissues.

**Figure 1:**
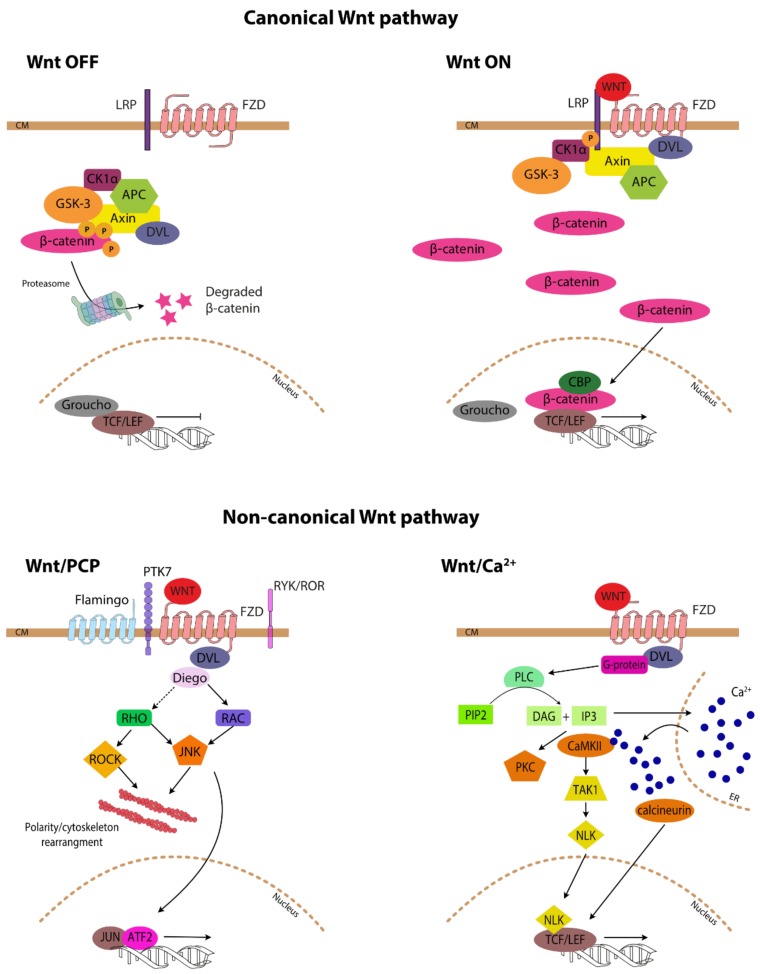
Canonical and non-canonical Wnt signalling.

Three distinct signalling cascades have been described: one referred to as canonical and two non-canonical (Figure 1). The canonical Wnt pathway becomes engaged upon binding of secreted Wnt proteins (e.g., Wnt1, Wnt3a) to a surface receptor complex consisting of Frizzled (FZD) receptors and lipoprotein receptor-related protein (LRP) co-receptors. LRP receptors are then phosphorylated by casein kinase 1 alpha (CK1α) and glycogen synthase kinase 3 beta (GSK3β), which recruits Dishevelled (DVL) proteins to the plasma membrane where they polymerise and become activated. In the absence of Wnt, β-catenin, the key effector molecule of the canonical pathway, is inactivated by the so-called “destruction complex”, consisting of axin, adenomatous polyposis coli (APC), GSK3β and CK1, which phosphorylates β-catenin leading to its proteasomal degradation. The DVL polymers inactivate the destruction complex resulting in the accumulation of unphosphorylated active β-catenin. Unphosphorylated β-catenin translocates into the nucleus where it displaces transcriptional repressors (e.g., TLE/Groucho) and binds to the members of the T-cell factor/lymphoid enhancer-binding factor (TCF/LEF) transcription factor family, thereby converting them from transcriptional repressors into activators [8]. In addition, β-catenin recruits transcriptional coactivators, e.g., cAMP response element binding (CREB)-binding protein (CBP), and other members of the transcriptional machinery and histone modifiers to form an active transcriptional complex [9].

The non-canonical planar cell polarity pathway (Wnt/PCP) regulates the cytoskeleton and thus impacts on cellular shape and migration, while the non-canonical Wnt/calcium (Wnt/Ca^2+^) pathway regulates calcium levels inside the cell [10]. The main difference between the canonical and non-canonical pathways is the independence from β-catenin of the two latter cascades although the distinction is not clear cut and some of the proteins formally linked to non-canonical Wnt signalling influence the canonical pathway. Non-canonical Wnt/PCP ligands bind to FZD receptors associated with diverse co-receptors (e.g., pseudo tyrosine kinase 7 (PTK7), receptor-like tyrosine kinase (RYK), receptor tyrosine kinase-like orphan receptor 1 (ROR1/2), Flamingo) and cytoplasmic adaptors (DVL, Prickle planar cell polarity protein, Diego) distributed asymmetrically upon the cell surface. Wnt/PCP signals are converted into actin cytoskeletal dynamics by signalling through Ras-related C3 toxin substrate (RAC) and Ras homologous (RHO) G-proteins. RHO activates RHO-associated kinase (ROCK), which is one of the major regulators of the cytoskeleton. Signalling through RAC and RHO results also in JNK-dependent transcriptional activation [11]. Wnt/Ca^2+^ signalling induces cytosolic

Ca^2+^ increase by causing its release from the endoplasmic reticulum (ER) or influx from outside the cell. The Wnt/Ca^2+^ pathway is initiated by Wnt ligands engaging the FZD receptors, often without a co-receptor binding, and then DVL followed by G protein-induced activation of phospholipase C-beta (PLC-beta), which cleaves phosphatidylinositol 4,5-bisphosphate (PIP2) into 1,2-diacylglycerol (DAG) and inositol 1,4,5-triphosphate (IP3). IP3 causes the release of Ca^2+^ from ER while DAG and IP3 together activate protein kinase C (PKC), the calcium/calmodulin-dependent protein kinase type II (CaMKII)-TGF-beta-activated kinase 1 (TAK1)-Nemo-like kinase (NLK) pathway and calcineurin phosphatase and transcription activation. Genes activated as a result of signalling through the Wnt/Ca^2+^ pathway regulate cell fate and cell migration [12].

Wnt antagonists belong to small protein families, such as secreted Frizzled-related proteins (sFRP), Dickkopf (DKK), Wnt inhibitory factors (WIF), sclerostin domain containing 1 (Wise)/sclerostin (SOST), Cerberus, insulin-like growth factor binging protein (IGFBP), Shisa, trophoblast glycoprotein also known as Waif1, APC down-regulated 1 (APCDD1), and TRAB domain containing 2a also known as Tiki1 (Table 1) [13]. Their common feature is to antagonise Wnt signalling by competitively displacing certain WNT ligands from their receptors or preventing Wnt receptor maturation [13].

Deregulation of Wnt proteins and their downstream signal transduction components has been associated with various tumour types (for example, reviewed in [8,10]). Most notably, truncating mutations in the APC gene are found in up to 85% of colorectal cancer cases [10]. Last but not least, Wnt signalling is also one of the effector pathways involved in the epithelial-to-mesenchymal transition (EMT) [14]. EMT is physiologically relevant during organogenesis and is chiefly pertinent to cancer progression and metastasis.

## 3. Wnt Signalling in the Pathogenesis of Acute Myeloid Leukaemia (AML)

### 3.1. Canonical Wnt Signalling

**Figure 2:**
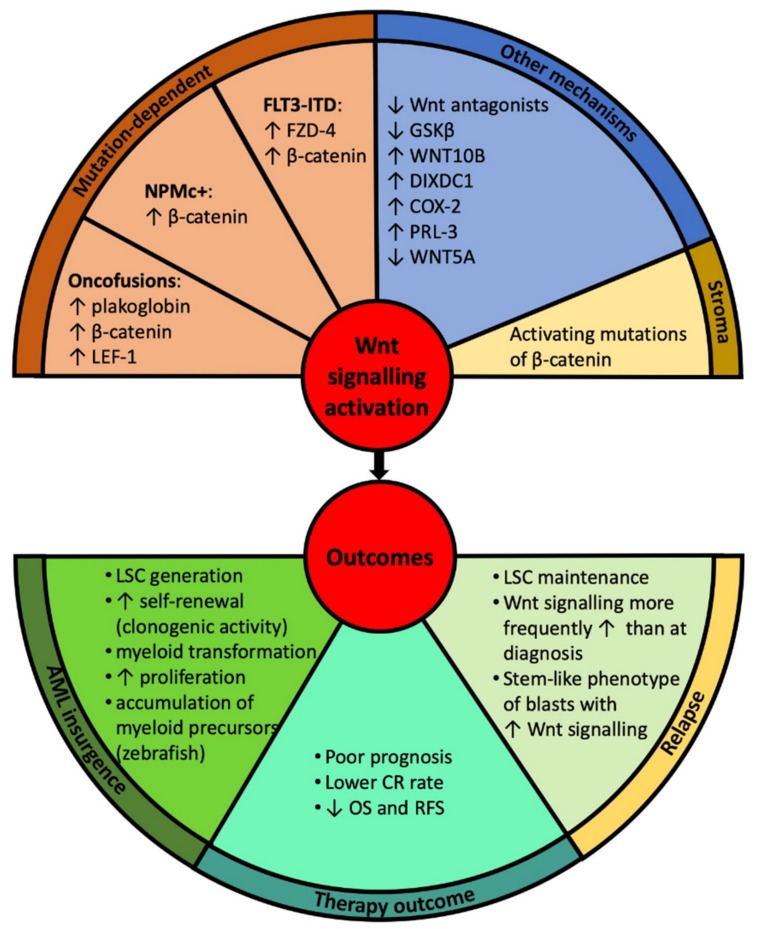
Mechanisms activating Wnt signalling in acute myeloid leukaemia (AML) and their consequences for the disease outcome.

**Figure 3:**
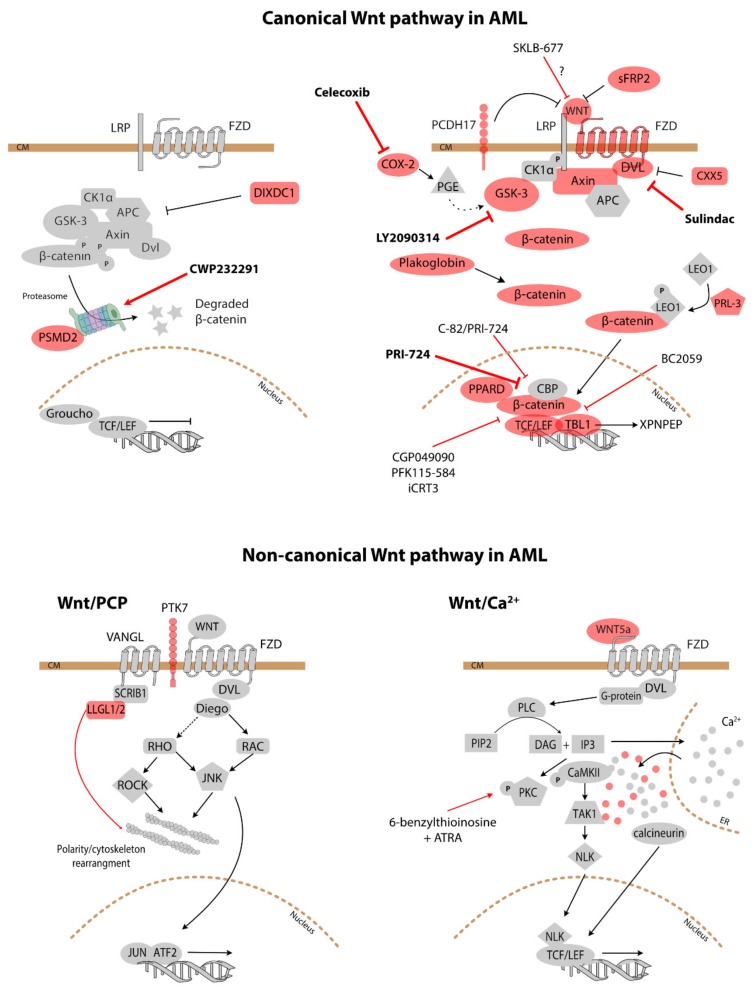
Wnt signalling in AML. The proteins in coral red have been specifically implicated in AML. Thin red bar-headed arrows indicate where investigational inhibitors act, while thick red bar-headed arrows show the targets of agents used in clinical trials. ER—endoplasmic reticulum.

Activation of Wnt signalling is a frequent although heterogenous feature of AML; Wnt signalling activation has been found to be present or absent even within cohorts characterised by the same genotype. Using transfection of a TCF/LEF reporter construct into primary AML cells, increased reporter activity was found in 16/25 leukaemia samples [15]. Moreover, a significant proportion of AML cases show aberrant expression of the Wnt pathway components such as WNT1, WNT2b and LEF-1 [15]. There is evidence for the involvement of the Wnt/β-catenin pathway in the pathogenesis of AML through a number of mechanisms that will be discussed here. Figure 2 summarises the mechanisms of Wnt signalling activation in AML and its outcomes, while Figure 3 maps the Wnt-related proteins and targets implicated in AML.

*Translocation products.* Translocation products, such as PML/RARα, AML1/ETO or PLZF1/RARα regulate genes associated with Wnt signalling [16]. Shared target genes of these fusion proteins have been identified in transfected U937 cells using high-density gene expression microarray analyses. Several of the genes regulated by all three fusion proteins were associated with Wnt signalling. γ-catenin, also known as plakoglobin, was one of the positive transcriptional regulators strongly induced on the mRNA and protein level by all three fusion proteins in U937. Its promoter was cloned and shown to be induced by AML1-ETO in 32D cells [17]. Primary blast cells carrying one of the fusion proteins also significantly overexpressed plakoglobin in vivo [16]. The crucial role of plakoglobin in increasing the self-renewal of haematopoietic stem cells (HSCs) upon expression of AML-associated translocation products was demonstrated by the abrogation of replating efficiency upon depletion of plakoglobin and its increase upon plakoglobin overexpression. Irradiated recipient mice injected with plakoglobin-transduced HSCs developed AML. These data provided evidence that the aberrant activation of Wnt signalling by the translocation products decisively contributes to the pathogenesis of AML [17].

Plakoglobin expression was associated with β-catenin stabilisation and nuclear localization both in AML blasts and in ectopic plakoglobin expression settings [18]. Two mechanism explaining the role of plakoglobin in Wnt signalling activation in AML have been proposed. First, β-Catenin knockdown demonstrated that plakoglobin induces TCF-dependent transcription. Interestingly, normal cells exclude plakoglobin and β-catenin from the nucleus, a level of regulation that is lost in the majority of AMLs. Second, the stability of both proteins is regulated by the destruction complex. High levels of plakoglobin may saturate the system and indirectly lead to β-catenin stabilisation [18].

More recently, it has been proposed that Wnt signalling induces the expression of AML1 and ETO genes, enhances their spatial proximity and generates translocation events [19].

LEF-1, the transcriptional factor of β-catenin is frequently overexpressed in AML [20,21,22]. Primary AML samples with t(8;21) or t(15;17) express higher levels of LEF-1 than those without either translocation [20] although another study found high LEF-1 expression also in a subset of cytogenetically normal AML patients [21]. Ectopic expression of wild-type LEF-1 or of a constitutively-active Lef-1 mutant in murine bone marrow led to the onset of AML in transplanted mice [23]. Moreover, LEF-1 determines the nuclear localisation of β-catenin [22]. The relative levels of nuclear LEF-1 and β-catenin are tightly correlated in both cell lines and in primary AML blasts as found in a β-catenin interactome mass spectrometry screen [22].

Further evidence for the crucial importance of Wnt signalling in the pathogenesis of AML was provided by experiments involving the MLL/ENL oncofusion, HOX9a and MEIS1 oncogenes. Wnt signalling was shown to be required for *HOX* gene- and MLL/ENL-driven transformation of haematopoietic stem/progenitor cells as AML did not form in the absence of β-catenin [24]. Indeed, Wnt/β-catenin signalling pathway is required for self-renewal of LSCs derived from either HSCs or more differentiated granulocyte macrophage progenitors [24]. A study of gene expression networks in AML LSCs derived from patients with primary and secondary AML (de novo and relapsed) confirmed that Wnt signalling was activated in LSCs compared to HSCs. Upregulated Wnt genes in LSCs included *axin*, *CK2* and *APC*, while *c-Jun* and *WIF1* were downregulated [25].

*NPM1.* We found that the leukaemogenic NPMc+ mutant activates canonical Wnt signalling during zebrafish development by causing an expansion of the haematopoietic progenitor pool in primitive zebrafish haematopoiesis. Wnt-signalling activation was indeed responsible for the myeloproliferative phenotype, since it was rescued by the overexpression of the dkk1b Wnt inhibitor. In addition, we established that canonical Wnt signalling is active in the patient derived OCI-AML3 cell line bearing NPMc+ and in AML blasts expressing NPMc+ [26].

In a study concerning clonal evolution patterns in AML with NPMc+, a series of 129 paired diagnosis-relapse samples were analysed [27]. Eleven patients lost the NPMc+ mutation at relapse and showed a diverse pattern of mutations and signalling pathway activation as uncovered through whole exome and RNA sequencing of paired samples. In a proportion of NPMc+ persistent cases, Wnt signalling was active in both diagnostic and relapse samples. By contrast, for NPMc+ loss patients, Wnt signalling was absent at presentation, but activated at the disease recurrence. A possibility exists that NPM1c+ loss samples showed less stem cell-like phenotypic features at diagnosis as underscored by the absence of the enrichment of the Wnt signalling, a key transduction pathway in the biology of LSCs, the therapy was successful in terms of total leukaemia eradication and that the relapse represented, in fact, a second de novo or treatment-related AML [27]. AML blasts from the majority of cases with NPMc+ mutation show morphological signs of differentiation and the CD34-CD33+ phenotype associated with common myeloid progenitors rather than with stem cells supporting the notion of dichotomous Wnt activation in this AML subtype [28,29].

*FLT3.* Activating FLT3 mutations occur in about 30% of patients with AML. The interaction between oncogenic FLT3-ITD mutations and Wnt signalling was studied in the myeloid progenitor cell line 32D [30]. Higher mRNA expression of FZD-4 receptor in 32D/FLT3-ITD than in 32D/FLT3-WT cells was found by microarray analysis, verified by real-time polymerase chain reaction (RT-PCR) and Western blotting. 32D/FLT3-ITD cells, compared to the control, showed increased β-catenin protein levels, irrespective of their exposure to Wnt3a ligand. Moreover, 5/7 AML samples with FLT3-ITD mutations expressed high β-catenin protein levels, whereas patients with wild-type FLT3 did not. FLT3-ITD induced TCF-dependent transcription, e.g., an increase of Wnt target gene mRNAs. In the presence of FLT3-WT or FLT3-ITD signalling, Wnt3a increased slightly 32D cell proliferation. The results indicate that FLT3-ITD and Wnt-dependent signalling pathways synergise in myeloid transformation [30].

*Other mechanisms of Wnt signalling activation.* Valencia et al. [31] found that Wnt activation is achieved in AML through methylation of Wnt antagonists. A methylation-specific polymerase chain reaction approach was used to analyse the promoter methylation status of the sFRP1, sFRP2, sFRP4, sFRP5, DKK1 and DKK3 Wnt antagonists. Aberrant methylation of Wnt antagonists was detected in up to 64% of AML marrow samples [31]. In the case of AML with t(8;21), the fusion protein binds to the sFRP1 promoter and represses its transcription via a consensus AML1-bining site [32]. Treatment of AML cell lines with 5-aza-2′-deoxycytidine, a demethylating agent, and the resulting re-expression of Wnt antagonists led to an inactivation of the Wnt pathway as detected through the Wnt pathway genes (e.g., *cyclin D1*), TCF1 and LEF1 downregulation and by the reduction of nuclear localization of β-catenin [31].

GSK3α/β removal lead to the occurrence of an aggressive AML. The canonical Wnt pathway was the most prevalently activated signalling cascade in the LSCs of GSK3α/β double knock-out mice. In this setting, Wnt signalling together with AKT and mTOR were responsible for the onset of AML [33].

A genetic rearrangement in blast cells involving intron 1 (IVS1) of the WNT10B locus flanked at the 5′ end by non-human DNA represents yet another mechanism of Wnt activation [34]. The resulting WNT10B^IVS1^ transcript was expressed mainly in intermediate/unfavourable risk AML patients [34]. The establishment of a primary cell culture of AML cells expressing AC133 stem cell marker demonstrated that leukaemia cells synthesise and secrete WNT10B ligand, a HSC regenerative-associated molecule, in response to which the levels of dephosphorylated β-catenin in vivo rise [35]. This suggests that AC133(bright) LSCs are promoted by misappropriating homeostatic haematopoietic regeneration Wnt programmes [35]. In addition, the expression of WNT10B in zebrafish embryos promotes the accumulation of haematopoietic precursors [34].

A recent report indicated that Wnt signalling might be upregulated in AML through the overexpression of dishevelled-axin domain containing 1 (DIXDC1) protein [36]. DIXDC1, involved in the progression and development of various tumours, is a scaffold protein shown to be highly expressed in AML cell lines and primary blasts but not in normal counterpart cells. It activates TCF-dependent transcription of Wnt target genes by interacting with DVL and axin [36].

Cyclooxygenase-2 (COX-2) is a key enzyme in the prostaglandin production that has been implicated in cancer [37]. COX-2 activates Wnt signalling through the prostaglandin E. A variety of oncogenes can induce expression of COX-2 in haematopoietic cells, indeed, circulating AML blasts express COX-2 [38].

Phosphatase of regenerating liver-3 (PRL-3) also known as protein tyrosine phosphatase 4A3 (PTP4A3) promotes key steps of cancer metastasis [39]. PRL-3 is expressed in 43% of AML samples, absent in normal haematopoietic cells [40] and implicated in LSC formation [41]. LEO1, a direct and specific substrate of PRL-3, binds to β-catenin, promoting its nuclear accumulation and transactivation of downstream target genes such as *c-myc* and *cyclin D1* [42]. AML cells expressing PRL-3 displayed increased sensitivity to β-catenin inhibition implying their addiction to Wnt signalling [42].

*Cell-extrinsic mechanisms.* HSCs and LSCs inhabit haematopoietic niches that are composed of a variety of cell types including osteoblasts, macrophages, mesenchymal stromal-, endothelial-, CXCL12-abundant reticular-, non-myelinating Schwann cells and sympathetic neurons that influence HSCs/LSCs both by direct contact and through secreted molecules [43]. Early studies demonstrated that Wnt signalling regulated haematopoiesis through stromal cells as Wnt3a-conditioned medium did not affect the generation of myeloid and B-cells in their absence [44]. However, LSCs, but not preleukaemic LSCs, activate cell-intrinsically Wnt signalling, rendering them independent of niche-derived signals and constraints applying to HSCs [45]. Curiously, AML can be induced by activating mutations in the β-catenin gene in osteoblasts [46]. Activated β-catenin stimulates expression of the Notch ligand, Jagged-1, in osteoblasts. Subsequent activation of Notch signalling in haematopoietic progenitors induces the transformation through altered differentiation potential. At the molecular level, FoxO1 interacts with β-catenin in osteoblasts to induce Jagged-1 expression [47]. Nuclear accumulation and increased β-catenin signalling in osteoblasts were detected in 38% of patients with myelodysplastic syndrome/AML [46]. Human bone marrow-mesenchymal stem cells induce galectin-3 expression in Kasumi-1 cell line and in primary AML blasts when co-cultured subsequently promoting GSK3β phosphorylation and β-catenin stabilization [48]. Adhesion of AML blasts to E-selectin expressed by endothelial cells in the vascular niche enhances their survival by Wnt activation as seen through quantitative PCR arrays validated by a Wnt-reporter assay performed on cells from 40 AML patients grown on E-selectin coated plates [49]. Vascular niche E-selectin induces mesenchymal-epithelial transition and Wnt activation in solid cancer cells [50].

### 3.2. Non-Canonical Wnt Signalling

*Non-canonical planar cell polarity pathway* WNT5A is considered an almost “purely” non-canonical Wnt ligand. Mice heterozygous for Wnt5a develop chronic myeloid leukaemia or B-cell disorders, while the analysis of 10 AML samples revealed deletion of the WNT5A gene and/or loss of WNT5A expression in a fraction of samples. The two notions suggest that Wnt5a suppresses hematopoietic malignancies [51].

Recent data suggest that Scribble 1 (SCRIB1) polarity complex, consisting of SCRIB1, VANGLE1 and lethal-giant-larvae 1 and 2 (LLGL1/2) proteins is involved in HSC polarity and AML biology. In mice, Llgl1 knockout leads to an expansion of the HSC pool, albeit without any evidence of AML development. A *Llgl1^−/−^* gene signature identified upon conditional deletion of *Llgl1* in mice was found in circa 25% of AML patients [52]. *Llgl2* mutations, instead, seem to be important in the transformation of severe congenital neutropoenia to AML [53].

The pseudo kinase receptor 7 (PKT7) is a pseudo kinase involved in the planar cell polarity pathway through the interaction with the FZD-DVL complex. PTK7 is expressed in ~70% of AML patients [54], while Ptk7-deficient mice have a decreased pool of HSCs resulting from aberrant proliferation and migration [55]. In AML, PTK7 plays a role in cell migration and increases resistance to apoptosis [54].

*Non-canonical Wnt calcium pathway* Zang et al. showed that 6-benzylthioinosine- and all-trans retinoic acid (ATRA)-induced differentiation of HL60 cells occurs through activation of the non-canonical Wnt/Ca^2+^ signalling pathway as treatment caused an elevation of intracellular calcium levels and led to the phosphorylation of CaMKII and PKC. The authors went on to analyse the expression levels of 4 genes: *FZD4*, *FZD5*, *WNT5a* and *RHOU* in the bone marrow mononuclear cells from 30 newly diagnosed AML patients and found their significant downregulation compared to normal controls and complete remission samples [56]. However, strict assignment of FZD receptor and Wnt ligand pairs to canonical versus non-canonical signalling is hard as the same receptors and ligands may mediate either pathway.

## 4. Clinical Implications—Prognosis and Therapeutic Approaches

### 4.1. Prognostic Impact

Several molecules involved in or modulating Wnt signalling have a prognostic value in AML (Table 2). Following the discovery of Wnt pathway activation in AML, it was ascertained that a high quantity of β-catenin was of prognostic significance in the disease. High levels of β-catenin expression in AML detected in Western blot analysis were found to be predictive of poor event-free survival (EFS), shortened overall survival (OS) and enhanced clonogenic activity [57]. Similarly, the finding of nuclear non-phosphorylated β-catenin occurrence by immunohistochemistry was associated with low complete remission (CR) rate and poor prognosis [58]. The prognostic role of β-catenin was further confirmed and its high expression levels linked to unfavourable-risk karyotypes in a study that retrospectively analysed a cohort of newly-diagnosed patients by immunohistochemistry [59]. Nuclear localization of β-catenin resulted more frequent in AML patients at relapse in paired diagnosis/relapse samples, with the caveat of an extremely small cohort analysed [60]. Similarly, plakoglobin expression in AML patients has a clinical significance [61]. Plakoglobin expression levels were significantly higher in AML patients with lower white blood cells (<30 × 10^9^/L) than in those with higher white blood cells (≥30 × 10^9^/L) and in AML patients with mutated *CEBPA*. AML patients with lower plakoglobin levels were more likely to achieve CR compared with patients with high plakoglobin levels [61].

High expression of LEF1 has been identified as a favourable prognostic factor in cytogenetically normal acute myeloid leukaemia [21]. Such a counterintuitive finding was confirmed by a further study, which determined that a high LEF-1 level was associated with better OS in patients with intermediate-risk cytogenetics and a favourable relapse-free survival in patients with unmutated FLT3 [20]. However, it should be stressed that high LEF1 levels are not necessarily equivalent to Wnt signalling activation, as LEF1 performs Wnt-independent functions, e.g., plays a role in granulocyte differentiation [62].

Patients with high levels of phosphorylated GSK3 (i.e., inactive form) had a shorter OS compared to those with low levels of this phospho-kinase in the total AML population [63]. High levels of phospho-GSK3 (pGSK3) correlated with adverse OS and lower CR incidence. This held true for patients with intermediate-, but not for those with unfavourable cytogenetics. Patients with intermediate cytogenetics and FLT3-ITD also displayed a better outcome when pGSK3 was low compared to counterparts with high levels of pGSK3 [63].

The prognostic significance of epigenetic regulation of Wnt pathway components in AML was studied by Valencia et al. In a subgroup of patients 60 years of age or younger with newly diagnosed AML and intermediate-risk cytogenetics, abnormal methylation of Wnt antagonists was associated with decreased 4-year relapse-free survival (28 vs. 61%, respectively, *p* = 0.03). Recently, an integrated computational approach of gene expression and DNA-methylation profiles of Wnt signalling genes was used to search for novel prognostic markers in AML [64]. Three novel prognostic factors (PSMD2, PPARD, XPNPEP) for OS and EFS were identified and a further four (LEF1, sFRP2, RUNX1, AXIN2) confirmed, all but PSMD2 and PPARD being negative prognostic factors [64]. Also, the epigenetic regulation of non-canonical Wnt signalling seems to have a prognostic value: WNT5A, a putative tumour suppressor gene in AML, is silenced by methylation in this disease that leads to the upregulation of cyclin D1 expression and confers poor prognosis in AML patients [65].

Protocadherin 17 (PCDH17) functions as a tumour suppressor downregulating Wnt/β-catenin signalling and cell metastasis and is frequently methylated in breast cancer [66]. Low PCDH17 expression is an independent, poor prognostic factor in AML and has an additional prognostic value in stratifying molecularly defined subgroups. Furthermore, low PCDH17 expressing patients were characterised by a gene-expression signature reflecting the deregulation of EMT- and Wnt pathway-related genes. Such deregulations likely contribute to the adverse outcome observed in patients with low PCDH17 levels [67].

A surprising and not fully understood prognostic association was made between the levels of CXXC-type zinc finger protein 5 (CXXC5) and AML. CXXC5 inhibits Wnt signalling and is a candidate tumour suppressor in AML [68,69,70]. However, despite the clear attenuation of Wnt signalling induced by CXXC5, it is its downregulation in primary patients’ samples that predicts a better prognosis in AML. Indeed, patients with low CXXC5 had a lower relapse rate and better OS and EFS at 5 years, independent of cytogenetics risk groups or other prognostic factors. Moreover, its low expression was associated with known prognostic factors such as frequency of *MLL* rearrangements, t(8;21) and lower frequency of *FLT3* mutations [68,69,70].

Of the non-canonical Wnt signalling pathway mediators, the *LLGL1* gene and PTK7 protein expression levels have a prognostic value in AML. Reduced *LLGL1* expression was associated with significantly decreased OS patients [52], while high expression of PTK7 by flow cytometry in AML correlated with core-binding factor aberrations (t(8;21), inv(16); 19/20 cases) and was also common in AML with 5q- and -7 (21/24 patients) or in cases with normal karyotype (56/65 patients), so in low, intermediate and high cytogenetic risk groups. PTK7-positive leukaemias showed low circulating blast counts, were more resistant to anthracycline-based frontline therapy and showed a reduced relapse-free survival [54].

### 4.2. Treatment

The rationale for targeting Wnt signalling seems obvious given that it is prominently involved in a number of processes that contribute to leukaemogenesis as shown above. Many of the studies published to date are still at the level of preclinical feasibility testing of Wnt inhibition in AML. Figure 3 illustrates the known compounds modulating Wnt signalling.

*Preclinical investigations.* The effect of CGP049090 and PFK115-584, two compounds that specifically inhibit the binding between β-catenin and LEF1, was evaluated in Kasumi-1 and HL-60 AML cell lines, primary AML blasts and healthy peripheral blood mononuclear cells as a control. The treatment resulted in a significant apoptosis of AML cell lines and primary blasts, but not of control cells [71].

To circumvent the Wnt inhibitor promoter methylation that is common in AML [31], a sequential combination of decitabine, a demethylating agent, and idarubicin was used. The combined treatment induced synergistic cell death of U937, HEL, SKM-1 cell lines and primary AML blasts. Subcutaneous tumour growth inhibition exerted by the sequential combination was higher than that of the single agent or control treatment in vivo. The Wnt pathway activation abrogation upon treatment was confirmed in both AML cell culture and animal studies through the demethylating of Wnt inhibitors [72].

The efficacy of combined therapy with the BC2059 β-catenin antagonist and histone deacetylase inhibitor was determined in AML cell lines [73]. BC2059, an anthraquinone oxime-analogue, is known to attenuate β-catenin levels through the disruption of its binding to the transducin β-like 1 (TBL1) scaffold protein and subsequent proteasomal degradation. BC2059 treatment induced dose-dependent apoptosis in cultured and primary AML blasts. Treatment with BC2059 also significantly improved the median survival of immunodeficient mice engrafted with either cultured or primary AML blasts. Co-treatment with panobinostat, a pan-histone deacetylase inhibitor, and BC2059 synergistically induced apoptosis of cultured and primary AML blasts and further improved the survival of mice, underscoring the promising potential of BC2059 [73].

SKLB-677, a novel FLT3 inhibitor was found to repress Wnt signalling, in contrast to the known first and second generation FLT3 inhibitors [74]. AML cell lines and primary AML blasts bearing FLT3 mutation showed higher sensitivity to SKLB-677 than AML with wild-type FLT3 or solid tumour cell lines. The compound considerably suppressed LSCs in vitro [74]. A similar result was obtained when combining C-82/PRI-724, a small molecule antagonist of the β-catenin/CBP complex, with sorafenib or quizartinib FLT3 inhibitors [75]. The inhibition of Wnt signalling suppressed AML cell growth, induced apoptosis, abrogated stromal protection, and synergised with FLT3 inhibitors in FLT3-mutated AML cells and stem/progenitor cells in vitro. The combination also improved the survival of AML-xenografted mice and impaired leukaemia engraftment. β-catenin inhibition abrogated the protection of the leukaemic stem/progenitor cells provided by the microenvironment [75].

COX-2 inhibitors, such as celecoxib, have been tested on a panel of AML cell lines and primary AML blasts. The effect of the combined celecoxib and doxorubicin treatment on the HL60 cell line and in primary AML blasts has also been analysed [76]. Both celecoxib alone and the combination induce apoptosis in leukaemic cell lines and AML blasts [38,76,77].

A less-encouraging picture emerged from the study by Griffith et al. [60]. The authors used multispectral imaging flow cytometry to identify AML samples with nuclear localization of β-catenin, a hallmark of Wnt activation, in order to select patients most likely to benefit from pharmacological Wnt inhibition. Primary samples were treated in vitro with iCRT3, a compound that blocks the interaction between β-catenin and TCF. Although, de novo AML samples with nuclear β-catenin responded the best in terms of cell death upon treatment, both differentiated and CD34+ cell populations increased in iCRT3-treated samples [60].

*Clinical trials.* Relatively few clinical trials concerning the use of Wnt signalling inhibitors/modifying molecules have been conducted, most of them in the initial (I, II) phases (Table 3). Of the five trials registered in the ClinicalTrials.gov database (accessed 10/08/2019) of privately and publicly funded clinical studies conducted around the world, the results are available for just one and are not encouraging. Indeed, although the treatment was found to be tolerable, no complete or partial remissions were recorded. There is hope in the trial that has been posted in March of the current year, in which the treatment will be tailored to the mutational status encountered. One of the combinations to be explored in the new trial involves the use of the aforementioned celecoxib.

## 5. Conclusions

The role of Wnt signalling in the pathogenesis of AML is clear and has been linked to both initiation and progression of AML and it is required for LSCs’ survival and self-renewal. Although, there are no doubts regarding the activation of Wnt signalling in AML, one should bear in mind that many, especially early studies, used as a readout the expression of genes that are not specific to Wnt signalling (e.g., *c-myc*, *cyclin D1*), i.e., regulated by a myriad of factors. In the era of next-generation sequencing, such an issue has ceased to be a problem. Wnt signalling in AML is deregulated at all levels of signal transduction: receptors, ligands, intermediate signal transducers, β-catenin stabilisation and nuclear translocation and transcription activation. Although a lot less studied, also the non-canonical pathways are deregulated in patients with this malignancy.

AML remains a deadly disease for which current treatments are unsatisfactory [78]. In the era of targeted therapies, no opportunities for disease cure must be missed. Wnt signalling continues to be explored as no single satisfactory Wnt-signalling inhibition exists. The Wnt-signalling pathway has always proved difficult to target because of its complexity, the variety of outcomes as well as cross-talk with other signalling pathways. There are questions that await an answer. What exactly should be inhibited? The receptors? β-catenin stabilisation/nuclear translocation? LEF/TCF-dependent transcription? Is Wnt inhibition going to be a universal therapeutic approach in AML, or do we need to select the right patient population that will most likely benefit from it? Multiple strategies for targeting Wnt signalling—ranging from small molecules to blocking antibodies, and peptide ago-/antagonists—are being developed and researched, thus paving the way for future clinical trials in cancer patients. In particular, there are hopes that Wnt inhibition could provide a way of eradicating LSCs.

Taken together, although the modulation in Wnt signalling may offer an important opportunity for future targeted therapy in AML, further work is warranted.

## Figures and Tables

**Table 1:** Wnt antagonists.

Wnt Antagonist	Name	Target	Mechanism of Action	Canonical/Non-Canonical Signalling Inhibition
Secreted	sFRP	Wnt	Sequestration of Wnt	Both
		FZD	Binding to FZD receptor	
	DKK 1, 2 and 4	LRP6	Disruption of FZD-LRP6 complex	Canonical
			Induction of LRP6 endocytosis	
	WIF	Wnt	Sequestration of Wnt	Both
	Wise/SOST	LRP6	Disruption of FZD-LRP6 complex	Canonical
	Cerberus	Wnt	Sequestration of Wnt	Both
	IGFBP	LRP6 and FZD	Disruption of FZD-LRP6 complex	Canonical
Trans-membrane	Waif1	LRP6	Disruption of FZD-LRP6 complex	Canonical
	APCDD1	Wnt and LRP5	Sequestration of Wnt	Canonical
	Tiki1	Wnt	Removal of eight amino-terminal residues from Wnt	Both

**Table 2:** Wnt signalling and Wnt signalling-modifying or target molecules with prognostic value.

Prognostic Factor	Number of Cases	Age (Median)	AML Type	Therapy	Technology Used	Prognosis	Reference
High β-catenin	82	57	De novo	Induction + consolidation	WB	Poor	[57]
	54	53	De novo	Not specified	IHC		[58]
	59	59.6	De novo	Induction + anthracycline or low dose cytarabine	IHC		[59]
	21	65	De novo/relapse	Not specified	Flow cytometry		[60]
High LEF-1	101	47	De novo	Untreated	RT-qPCR	Good	[20]
	406	<60	De novo	Induction + consolidation	Microarrays and RT-qPCR		[21]
	344	48	De novo/relapse/secondary	Induction + consolidation ± transplantation	Genome-wide mRNA and DNA-methylation profiling data *		[64]
High phosphorylated-GSKβ	511	65.7	De novo	Not specified	Reverse-phase protein analysis	Poor	[63]
Low PSMD2	344	48	De novo/relapse/secondary	Induction + consolidation ± transplantation	Genome-wide mRNA and DNA-methylation profiling data *	Good	[64]
High PPARD						Good	
High XPNPEP						Poor	
Low sFRP2						Poor	
Hypermethylated RUNX1						Poor	
Low AXIN2						Poor	
Low WNT5A	252	57	De novo	Induction + consolidation ± transplantation	Methylation-specific PCR	Poor	[65]
Low PCDH17	626	52	De novo	Induction + consolidation ± transplantation	RT-qPCR	Poor	[67]
High CXXC5	27	64	De novo ^$#^	Induction + consolidation	RT-qPCR	Poor	[68]
	67	64	De novo/secondary ^$^	Not specified	RT-qPCR		[69]
Low CXXC5	529	46	De novo	Induction + consolidation ± transplantation	Microarray and RT-qPCR	Good	[70]
Low LLGL1	83	<60	Normal karyotype	Not specified	RT-qPCR	Poor	[52]
High PTK7	184	63	De novo/secondary ^$^	Induction + consolidation ± transplantation	Flow cytometry	Poor	[54]

*—bioinformatic approach, ^#^—plus two published cohorts, ^$^—unselected consecutive patients, IHC—immunohistochemistry, WB—Western blotting, RT-qPCR—real-time quantitative polymerase chain reaction.

**Table 3:** Clinical trials involving Wnt signalling inhibitory/modulating compounds.

Compound	Target	Clinical Trial Phase (Number)	Status	Results
Celecoxib	COX-2	Phase Ib (NCT03878524)	Not yet recruiting	NA
CWP232291	β-catenin degradation	Phase I (NCT01398462)	Completed	No results posted
LY2090314	GSK3β	Phase II (NCT01214603)	Completed	Well tolerated No CR or PR
PRI-724	β-catenin/CBP	Phase I (NCT01606579)	Completed	No results posted
Sulindac	PDZ domain of DVL	Phase II (NCT01843179)	Withdrawn (lack of funding)	No results posted

CR—complete remission; PR—partial remission.
